# Dihydrofolate-Reductase Mutations in *Plasmodium knowlesi* Appear Unrelated to Selective Drug Pressure from Putative Human-To-Human Transmission in Sabah, Malaysia

**DOI:** 10.1371/journal.pone.0149519

**Published:** 2016-03-01

**Authors:** Matthew J. Grigg, Bridget E. Barber, Jutta Marfurt, Mallika Imwong, Timothy William, Elspeth Bird, Kim A. Piera, Ammar Aziz, Usa Boonyuen, Christopher J. Drakeley, Jonathan Cox, Nicholas J. White, Qin Cheng, Tsin W. Yeo, Sarah Auburn, Nicholas M. Anstey

**Affiliations:** 1 Menzies School of Health Research and Charles Darwin University, Darwin, Northern Territory, Australia; 2 Infectious Diseases Society Sabah-Menzies School of Health Research Clinical Research Unit, Kota Kinabalu, Sabah, Malaysia; 3 Department of Molecular Tropical Medicine and Genetics, Faculty of Tropical Medicine, Mahidol University, Bangkok, Thailand; 4 Mahidol Oxford Tropical Medicine Research Unit, Mahidol University, Bangkok, Thailand; 5 Clinical Research Centre, Queen Elizabeth Hospital, Sabah, Malaysia; 6 Jesselton Medical Centre, Kota Kinabalu, Sabah, Malaysia; 7 Faculty of Infectious and Tropical Diseases, London School of Hygiene and Tropical Medicine, London, United Kingdom; 8 Royal Darwin Hospital, Darwin, Northern Territory, Australia; 9 Lee Kong Chian School of Medicine, Nanyang Technological University, Singapore, Singapore; 10 Australian Army Malaria Institute, Brisbane, Australia; 11 Clinical Tropical Medicine, Queensland Institute of Medical Research, Brisbane, Australia; Ehime University, JAPAN

## Abstract

**Background:**

Malaria caused by zoonotic *Plasmodium knowlesi* is an emerging threat in Eastern Malaysia. Despite demonstrated vector competency, it is unknown whether human-to-human (H-H) transmission is occurring naturally. We sought evidence of drug selection pressure from the antimalarial sulfadoxine-pyrimethamine (SP) as a potential marker of H-H transmission.

**Methods:**

The *P*. *knowlesi* dihdyrofolate-reductase (*pkdhfr*) gene was sequenced from 449 *P*. *knowlesi* malaria cases from Sabah (Malaysian Borneo) and genotypes evaluated for association with clinical and epidemiological factors. Homology modelling using the *pvdhfr* template was used to assess the effect of *pkdhfr* mutations on the pyrimethamine binding pocket.

**Results:**

Fourteen non-synonymous mutations were detected, with the most common being at codon T91P (10.2%) and R34L (10.0%), resulting in 21 different genotypes, including the wild-type, 14 single mutants, and six double mutants. One third of the *P*. *knowlesi* infections were with *pkdhfr* mutants; 145 (32%) patients had single mutants and 14 (3%) had double-mutants. In contrast, among the 47 *P*. *falciparum* isolates sequenced, three *pfdhfr* genotypes were found, with the double mutant 108N+59R being fixed and the triple mutants 108N+59R+51I and 108N+59R+164L occurring with frequencies of 4% and 8%, respectively. Two non-random spatio-temporal clusters were identified with *pkdhfr* genotypes. There was no association between *pkdhfr* mutations and hyperparasitaemia or malaria severity, both hypothesized to be indicators of H-H transmission. The orthologous loci associated with resistance in *P*. *falciparum* were not mutated in *pkdhfr*. Subsequent homology modelling of *pkdhfr* revealed gene loci 13, 53, 120, and 173 as being critical for pyrimethamine binding, however, there were no mutations at these sites among the 449 *P*. *knowlesi* isolates.

**Conclusion:**

Although moderate diversity was observed in *pkdhfr* in Sabah, there was no evidence this reflected selective antifolate drug pressure in humans.

## Introduction

Zoonotic transmission of *P*. *knowlesi* from its simian hosts to humans has likely been occurring since the human population first expanded in Southeast Asia around 40,000 years ago[1,2]. Despite microscopy misdiagnosis of *P*. *knowlesi* with other human *Plasmodium* species[3,4], accurate molecular detection has validated recent reports of an increasing incidence of *P*. *knowlesi* malaria cases in Eastern Malaysia[5–7], and the continued presence of low *P*. *knowlesi* endemicity in other countries in Southeast Asia[8–14]. The large geographical distribution of *P*. *knowlesi* is constrained only by the range of its natural macque hosts and *Leucosphyrus* group vector[15].

Human-to-human (H-H) transmission may also have been present since humans were first infected, with studies conducted in the 1960s demonstrating that *Anopheles balabacensis* (the most common malaria vector in Sabah, Malaysia[16]) can transmit *P*. *knowlesi* competently between human hosts[17]. Despite this, evidence to date suggests that *P*. *knowlesi* transmission remains primarily zoonotic. *P*. *knowlesi* circumsporozoite protein (CSP) gene and mitochondrial genome sequence data from studies in the Malaysian state of Sarawak[1], Peninsular Malaysia[18], Singapore[19] and Thailand[10] have found no evidence of divergence between the infections in human versus macaque hosts. In addition, ongoing *P*. *knowlesi* transmission has not been reported in areas where the long-tailed or pig tailed macaque hosts[20] are not present, consistent with recent modelling indicating a very low likelihood of sustained H-H transmission together with a low human reproductive rate (R_OH_ = 1.04)[21].

However, with an increasing incidence of knowlesi malaria in Malaysia[6], along with evidence of peri-domestic transmission[22], it is possible that H-H transmission is occurring to at least some degree. Molecular markers, such as monitoring for effects of selective antimalarial drug pressure on parasite populations, may provide a novel indicator, and would allow evaluation of spatio-temporal trends as seen in mapping of antimalarial drug-resistant genotypes in other areas [23,24]. Resistance to antifolate medications such as pyrimethamine arises readily in *P*. *falciparum*[25,26] and *P*. *vivax*[27–29] and has been well documented throughout Southeast Asia, including Malaysia[30,31]. In Malaysia, proguanil was deployed from the late 1940s, and resistance documented almost immediately. Pyrimethamine resistance was documented from the mid-1970s onwards[32], although sulfadoxine-pyrimethamine (SP) continued to be used as a first-line medication for the treatment and prophylaxis of *P*. *falciparum* for over 30 years until artemisinin-combination therapy (ACT) was introduced after 2009. The use of single dose pre-referral SP for falciparum malaria continues in remote areas of Sabah and Sarawak in Eastern Malaysia. As *P*. *knowlesi* is commonly misdiagnosed as *P*. *falciparum* due to morphological similarities in the early ring stages[3,7], and as co-infections of *P*. *knowlesi* with *P*. *falciparum* or *P*. *vivax* do occur[33], it is highly likely *P*. *knowlesi* parasite populations in humans would have been exposed to SP in Malaysia. Additional antifolate drug exposure from medications such as trimethoprim, used commonly for non-malarial infections, has demonstrated pyrimethamine cross-resistance in *P*. *falciparum*[34].

Pyrimethamine acts on *Plasmodium* parasites by inhibiting the dihydrofolate-reductase (*dhfr*) enzyme involved in the folate biosynthesis pathway. Non-synonymous point mutations in the *dhfr* gene affecting drug-enzyme binding confer resistance to treatment [25]. In *P*. *falciparum dhfr* (*pfdhfr*) this is manifest firstly with the acquisition of the S108N mutation, followed by increasing resistance associated with the addition of mutations located at residues 51, 59 and 164[35]. For *P*. *vivax dhfr* (*pvdhfr*) orthologous mutations to *pfdhfr* are located at codons 50, 58, 117, and 173 respectively [36] although these may not confer the same degree of functional resistance to pyrimethamine[37]. Sequencing of *pkdhfr* to date has only been reported from clinical isolates in the Andaman and Nicobar Islands in India, which have a very low *P*. *knowlesi* endemicity predominantly consisting of mixed infections with either *P*. *falciparum* or *P*. *vivax* [38]. Analysis revealed discordant mutations between *Plasmodium* species with only wild type *pkdhfr* reported together with multiple mutations of *pfdhfr* or *pvdhfr*, consistent with zoonotic-only transmission of *P*. *knowlesi* [38].

To assess selection pressure provided by antifolates as a possible surrogate marker of naturally occurring H-H transmission we undertook *P*. *knowlesi dhfr* sequencing in 449 human malaria cases in Sabah, Malaysia. As only human hosts would be expected to have SP drug exposure, the presence of similar functional *pkdhfr* mutation genotypes in spatio-temporal clusters would support the likelihood of H-H transmission. Sustained transmission of *P*. *knowlesi* genotypes with *dhfr* mutations would suggest that competent transmission from humans back to the monkey host reservoir is occurring naturally, and hence increase the possibility of H-H transmission. In addition it was hypothesised that clinical correlates of human adapted *P*. *knowlesi* parasites would be associated with *pkdhfr* mutations. Early neurosyphillis studies demonstrated repeated blood passage of *P*. *knowlesi* through humans resulted in higher parasite counts and risk of severe disease[39,40]. This is consistent with *P*. *knowlesi* adapting to invade a wider age range of human RBCs over time in an *in vitro* culture system[41], with hyperparasitaemia a known independent predictor of severe knowlesi malaria[42]. *P*. *knowlesi* invasion gene variants *Pknbpxa* and *Pknbpxb* have also been associated with increased risk of hyperparasitaemia and manifestations of severe disease[43]. A study also described an admixture of two distinct parasite sub-populations arising from the separate long-tailed and pig-tailed macaque hosts, including in samples from Sabah, with increased hybridisation between these two sub-populations potentially associated with parasite adaptation for humans[44]. Evaluating H-H transmission remains problematic but is important in the context of malaria elimination goals in South-East Asia, particularly due to the inability to manage the simian reservoir of *P*. *knowlesi* with conventional public health measures.

## Methods

### Study sites and patient enrolment

The study was conducted at Queen Elizabeth Hospital (QEH), Kudat District Hospital (KDH) and Kota Marudu District Hospital (KMDH). QEH is an adult tertiary-referral hospital located in Sabah’s capital city Kota Kinabalu, and serves as a referral hospital for the West Coast and Kudat Division of Sabah, comprising 6 district hospitals (including KDH and KMDH) with a catchment population of 1.14 million. Patients with severe malaria are generally referred from district hospitals to QEH. KDH and KMDH are located in adjacent districts in Kudat Division, northeast Sabah, with a combined catchment area of 3200 square kilometres and a population of 150,000. Details of the three study sites and referral practices have been described elsewhere [42,45].

Patients at QEH were enrolled from September 2010 –June 2014 alongside a prospective study involving consecutive non-pregnant patients ≥12 years old who were admitted with PCR-confirmed malaria, were within 18 hours of commencing malaria treatment, had no major co-morbidities or concurrent illness, and had not been previously enrolled[46]. For the current study, patients with a PCR-confirmed *P*. *knowlesi* monoinfection were included, in addition to 48 randomly selected patients with a *P*. *falciparum* monoinfection. At KDH and KMDH non-pregnant patients ≥1 year old admitted with PCR-confirmed knowlesi malaria were enrolled from December 2012 –June 2014 as part of separate prospective studies described in detail elsewhere[45,47].

### Ethics statement

This study was approved by the human research ethics committees of the Malaysian Ministry of Health (NMRR-10-754-6684), Menzies School of Health Research, Australia (HREC 2010–1431), and the London School of Hygiene and Tropical Medicine, U.K. (#6244). Written informed consent was provided by all patients, or if under the age of 18 years then by a parent or guardian.

### Study procedures

Baseline epidemiological and clinical information were recorded on standardised forms. Severe knowlesi malaria was defined according to modified WHO 2010 criteria for severe falciparum malaria, as previously described[46]. Pre-treatment blood slides were obtained on enrolment and read by an experienced research microscopist, with parasite counts per microlitre reported[48].

### PCR amplification and sequencing of *pkdhfr* and *pfdhfr*

DNA was extracted from 100 μL of packed red blood cell pellets using the QIAamp® DNA Blood Kit (Qiagen, Australia) according to the manufacturer’s instructions. *Plasmodium* species were confirmed as described previously[49,50]. A nested PCR approach using the same assay and cycling conditions for both primary and nested PCR was used to amplify *pfdhfr* and *pkdhfr* (i.e., 95°C for 3 min, followed by 25 cycles of 95°C for 30 sec, 52°C for 90 sec, and 72°C for 90 sec). The primary PCR was performed in a 50 μL reaction buffer volume, the nested PCR in a 100 μ L reaction buffer volume containing: 200 μM each deoxyribonucleotide triphosphate (dNTP; Bioline, Australia), 3 mM MgCl_2_, (Qiagen, Australia), 0.05U/ μL Taq polymerase (Qiagen, Australia), and 200 nM each primer pair (Life Technologies, Australia): a) *pfdhfr* primary reaction: *pfdhfr*-p-fw [5’-TTTATGATGGAACAAGTCTGC-3’] and *pfdhfr*-p-rev [5’-taaatgataaaatccaatgttgtat-3’]; b) *pfdhfr* nested reaction: *pfdhfr*-n-fw [5’-acaagtctgcgacgttttcgatatttatg-3’] and *pkdhfr*-n-rev [5’-agtatatacatcgctaacaga-3’]; c) *pkdhfr* primary reaction: *pkdhfr*-p-fw [5’-TTATGGAACAAGTTTCCGACG-3’] and *pkdhfr*-p-rev [5’-TAAATCACAAAGTCCAGAGTGGTGCCC-3’]; d) *pkdhfr* nested reaction: *pkdhfr*-n-fw [5’-TTTCCGACGTTTTCGATATTTACGC-3’] and *pkdhfr*-n-rev [5’-GTTGTACACCTCGCTGACCGA-3’]. The primary PCR was run using 2.5 μL DNA; 5 μL primary PCR was used in the nested PCR reaction. DNA sequencing of the nested PCR amplicons was performed by Macrogen (Seoul, Korea) [S1 File]. Sequences were analysed using Chromas Pro software (Technolysium Ltd., Australia) and BioEdit Sequence Alignment Editor[51] against the reference *P*. *knowlesi* genome[52]. Polyclonal infections, as represented by double peaks in the electropherogram, were observed in 7 infections (1x 34R/L 1x 52L/V, 1x 91P/A, 2x 91P/T, and 2x 149A/V) and categorized as mutant alleles[35].

### DNA sequence diversity and neutrality tests

DnaSP (version 5.10.01) was used to calculate the total number of polymorphic sites, singletons and haplotypes in the *pkdhfr* and *pfdhfr* fragments. Tajima’s neutrality test (D test) was also implemented with DnaSP software[53]. The nucleotide diversity at synonymous (*d*S), non-synonymous (*d*N), and all nucleotide sites (π) was calculated using the Nei-Gojobori method[54] with Jukes-Cantor correction[55] as implemented in the MEGA software (version 6.06). The standard error was determined by 1,000 bootstrap replications, and the rates of synonymous versus non-synonymous substitutions were compared using the Z-test of selection (MEGA 6.06).

### 3D *pkdhfr* protein modelling

*Pkdhfr* amino acid sequence was subjected to Basic Local Alignment Search (BLAST)[56], and further alignment was performed using ClustalW[57]. The sequence with maximum identity of 82.5%, *pvdhfr*, (PDB ID: 2BL9) was used as a template for homology modeling using SWISS MODEL server in http://swissmodel.expasy.org[58–60]. The constructed model was validated by PROCHECK[61]. The effects of both non-synonymous orthologous and nearby *pkdhfr* point mutations found in the current study on the pyrimethamine drug binding pocket were then evaluated by Autodock Vina[62].

### Mapping of geographic distribution of *pkdhfr* genotypes

All patients with knowlesi malaria enrolled at district sites had their household and central village locations determined using a hand-held global positioning system (GPS; Garmin^TM^; model 62sc) by local research fieldworkers. Patients enrolled at QEH had the central point of their village located and GPS coordinates recorded using Google Earth (version 7.1.2; Landsat images, July 2013), with assistance and corroboration via government public health and 2010 census maps, local fieldworkers, and input from patients where necessary. Final mapping of *pkdhfr* genotypes at village locations was performed using Quantum GIS software (version 2.4.0, www.qgis.org).

### Spatial and temporal clustering

Analysis of spatial, temporal and combined space-time clustering of *pkdhfr* genotypes was performed with SaTScan^TM^ software (version 9.2, http://satscan.org/), as previously described[60,63]. Data was inputted in a 0/1 event Bernoulli model with a case file including the number of patients with the specific *pkdhfr* genotype of interest at each village location and date, and a control file with the number, location and date of all other *pkdhfr* genotypes (the background distribution population). This software then uses the Kulldorf spatial[64], temporal or retrospective space-time scan statistic[65] to assess whether the specified *pkdhfr* genotypes are randomly distributed in space and/or time. Both large and small clusters are detected, with up to 50% of the study population allowed in a potential cluster within a maximum 30km radius. The time period for the temporal and space-time analysis was defined as 30 days, based on the average time of parasite maturation in the *An*. *leucosphyrus* vector (10 days)[66], the human pre-patent period (9–12 days)[17], and median fever duration before presentation (5 days) [46].

### Statistical analysis

Data were analysed using Stata^TM^ (version 12) software. Continuous variables were compared using Student *t* test or Mann-Whitney test depending on distribution, and proportions were compared using Chi squared or Fisher’s exact test. The Cuzick’s test for trend was used to assess the change in proportion of *pkdhfr* mutations over time, with enrolments at QEH (September 2010 –June 2014) divided into 5 x 9-month time-periods and enrolments at the district hospitals (December 2012 –June 2014) divided into 3 x 6-month time-periods.

## Results

### Study population

From September 2010 –June 2014, 504 patients with PCR-confirmed *P*. *knowlesi* monoinfection were enrolled at the 3 study sites, with *pkdhfr* sequence data available from 449 (89%) patients. Of those with *pkdhfr* sequence data, 263 (59%) patients were enrolled at QEH and 186 (41%) were enrolled at KDH/KMDH. Baseline demographics are summarised in **Table 1**. Overall, 78% of patients were male. Median age was 44 years at QEH adult hospital (range 14–94 years) and 30 years at KDH/KMDH (range 1–78 years). At KDH/KMDH, 40 (18%) children <12 years old were enrolled, with sequencing of the *pkdhfr* gene performed on 29 of these patients. From all study sites there were 190 (42%) farmers or plantation workers. Nearly all patients (96%) reported some forest or plantation exposure during the preceding 6 weeks, and 55% reported having seen a monkey during this time. Thirteen patients enrolled into the study at KDH/KMDH were subsequently transferred to QEH because of severe malaria. Sequencing of the *pkdhfr* gene was performed on all of these patients.

**Table 1:** Baseline features of patients with *pkdhfr* sequenced. Data are N (%) unless otherwise indicated. No significant differences (p<0.05) were seen between those with pkdhfr mutations and wild type.

**Patient characteristic**	***Pkdhfr* mutations**	***Pkdhfr* wild-type**
n (%) unless otherwise indicated	**(n = 145)**	**(n = 304)**
**Male sex**	113 (78)	232 (76)
**Age, years**		
Median (IQR)	36 (21–52)	41 (26–53)
Range	2–83	1–94
**Site of enrolment**		
QEH	81 (56)	182 (60)
KDH and KMH	64 (44)	122 (40)
**Occupation**		
Farmer	31 (21)	66 (22)
Plantation worker	33 (23)	60 (20)
Other	81 (56)	178 (59)
**Previous malaria (self-reported)**	75 (51)	135 (44)
**Reported forest/plantation exposure in past 6 weeks** ^#^	138 (95)	292 (96)
**Seen a monkey in past 4 weeks**	78 (54)	167 (55)
**Parasite count (parasites/**μ**l)**		
Median (IQR)	4680 (1363–28069)	3751 (688–16810)
**Severe malaria**	30 (21)	57 (19)
**Patient characteristic**	***Pkdhfr* mutations**	***Pkdhfr* wild-type**	
n (%) unless otherwise indicated	**(n = 145)**	**(n = 304)**	
**Male sex**	113 (78)	232 (76)	
**Age, years**			
Median (IQR)	36 (21–52)	41 (26–53)	
Range	2–83	1–94	
**Site of enrolment**			
QEH	81 (56)	182 (60)	
KDH and KMH	64 (44)	122 (40)	
**Occupation**			
Farmer	31 (21)	66 (22)	
Plantation worker	33 (23)	60 (20)	
Other	81 (56)	178 (59)	
**Previous malaria (self-reported)**	75 (51)	135 (44)	
**Reported forest/plantation exposure in past 6 weeks** ^#^	138 (95)	292 (96)	
**Seen a monkey in past 4 weeks**	78 (54)	167 (55)	
**Parasite count (parasites/**μ**l)**			
Median (IQR)	4680 (1363–28069)	3751 (688–16810)	
**Severe malaria**	30 (21)	57 (19)	

^#^ Includes living within 20 minutes’ walk of forest/plantation, or having spent >4 hours in a forest/plantation

### *Pkdhfr* mutations

The 651 bp fragment (spanning codon 1–217 of the *pkdhfr* gene) was sequenced successfully for 449/504 (89%) *P*. *knowlesi* samples. Seventeen non-synonymous mutations were detected, including 16 monomorphic and 1 polymorphic (T91P/A) mutation, with 145 (32%) patients having one or more mutations (**Table 2**). Among these, the most common were R34L and T91P, being detected in 45 (10.0%) and 46 (10.2%) patients, respectively. V149A was detected in 21 (4.7%) patients, and A44T, V52L, T91A, E119V and P129Q were each detected in 6 (1.3%) patients. Among patients with mutations detected, 131 (90%) had a single *pkdhfr* mutation and 14 (10%) had two mutations, with the most common double mutations being R34L-V149A, and T91A-P129Q, both seen in 4 (0.9%) patients, and T91P-D157Y, seen in 3 (0.7%) patients (**Table 3**). There were no significant differences in the most common mutations detected between patients enrolled at QEH compared to those enrolled at KDH or KMDH.

**Table 2:** *Pkdhfr* mutations among patients enrolled at Queen Elizabeth Hospital, and Kudat and Kota Marudu District Hospitals with knowlesi malaria.

		No. (%) of isolates from QEH^†^	No. (%) of isolates from Kudat/KM	Total
Amino acid position	Allelic variants	(n = 263)	(n = 186)	(n = 449)
24	D (wild type)	262 (99.6)	186 (100)	448 (99.8)
	Y (mutant)	1 (0.4)	0	1 (0.2)
29	E (wild type)	262 (99.6)	186 (100)	448 (99.8)
	K (mutant)	1 (0.4)	0	1 (0.2)
34	R (wild type)	232 (88.2)	172 (92.5)	404 (90.0)
	L (mutant)*	31 (11.8)	14 (7.5)	45 (10.0)
44	A (wild type)	262 (99.6)	181 (97.3)	443 (98.7)
	T (mutant)	1 (0.4)	5 (2.7)	6 (1.3)
52	V (wild type)	259 (98.5)	184 (98.9)	443 (98.7)
	L (mutant)	4 (1.5)	2 (1.1)	6 (1.3)
77	E (wild type)	262 (99.6)	185 (99.5)	373 (99.4)
	G (mutant)	1 (0.4)	1 (0.5)	2 (0.6)
91	T (wild type)	239 (90.9)	157 (84.4)	396 (88.2)
	P (mutant)*	21 (8.0)	25 (13.4)	46 (10.2)
	A (mutant)	3 (1.1)	3 (1.6)	6 (2.4)
	P/A (mutant)	0 (0)	1 (0.5)	1 (0.2)
92	H (wild type)	259 (98.5)	185 (99.5)	444 (98.9)
	Q (mutant)	4 (1.5)	1 (0.5)	5 (1.1)
108	Q (wild type)	262 (99.6)	186 (100)	448 (99.8)
	H (mutant)	1 (0.4)	0	1 (0.2)
119	E (wild type)	257 (97.7)	186 (100)	443 (98.7)
	V (mutant)	6 (2.3)	0	6 (1.3)
129	P (wild type)	259 (98.5)	184 (98.9)	443 (98.7)
	Q (mutant)	4 (1.5)	2 (1.1)	6 (1.3)
149	V (wild type)	256 (97.3)	172 (92.5)	428 (95.3)
	A (mutant)*	7 (2.7)	14 (7.5)	21 (4.7)
157	D (wild type)	260 (98.9)	184 (98.9)	444 (98.9)
	Y (mutant)	2 (0.8)	2 (1.1)	4 (0.9)
	N (mutant)	1 (0.4)	0	1 (0.2)
160	L (wild type)	263 (100)	185 (99.5)	448 (99.8)
	F (mutant)	0 (0)	1 (0.5)	1 (0.2)

*The most common mutations (prevalence >4%)

^†^Excludes the 13 patients enrolled into the study at KDH/KMDH and subsequently transferred to QEH.

**Table 3:** *Pkdhfr* genotypes among patients enrolled at QEH, Kudat District Hospital and Kota Marudu District Hospital.

Genotype	Frequency	Percent
Wild Type*	304	67.71
T91P	42	9.35
R34L	40	8.91
V149A	17	3.79
A44T	6	1.34
E119V	6	1.34
H92Q	5	1.11
V52L	5	1.11
R34L, V149A	4	0.89
T91A, P129Q	4	0.89
T91P, D157Y*	3	0.67
E77G	2	0.45
T91A	2	0.45
T91P/A	1	0.22
D157Y*	1	0.22
D24Y	1	0.22
E29K	1	0.22
L160F	1	0.22
P129Q	1	0.22
R34L, D157N*	1	0.22
T91P, P129Q	1	0.22
V52L, Q108H	1	0.22
**Total**	**449**	**100.00**

*Indicate genotypes for which corresponding protein structures were not modelled

### *Pfdhfr* mutations

The 613 bp fragment (spanning codon 1–204 of the *pfdhfr* gene) was successfully sequenced for 47/48 (98%) randomly selected *P*. *falciparum* positive samples from patients enrolled at QEH. Four non-synonymous, monomorphic mutations, known to be associated with pyrimethamine resistance (i.e., S108N, C59R, N51I, and I164L) were detected: Whereas the double mutant 108N+59R was fixed in the *P*. *falciparum* population, the triple mutants 108N+59R+51I and 108N+59R+164L were detected in 2/47 (4%) and 4/47 (8%) of *P*. *falciparum* infections, respectively.

### DNA sequence diversity and tests of neutrality

After excluding sequences with missing data, a total of 446 *pkdhfr* sequences and 45 *pfdhfr* sequences were available for analysis of sequence diversity. High levels of diversity were observed across the *pkdhfr* fragment, with 113 sites displaying evidence of single nucleotide variation within Sabah (**Table 4**). A total of 188 haplotypes were observed. The overall haplotype diversity (Hd) and nucleotide diversity (π) was estimated to be 0.983 and 0.006. The pairwise nucleotide diversity was significantly higher at synonymous (*d*S = 0.018) versus non-synonymous (*d*N = 0.003) sites (*p* = 0.007). The Tajima’s *D* value for the *pkdhfr* region was -2.24 (*p<*0.001), reflecting an excess of low frequency polymorphism relative to expectation.

**Table 4:** DNA sequence diversity in p*kdhfr* and *pfdhfr*.

Sequence	Residues	S^1^	H^2^	Hd^3^ (s.d)	π^4^(s.e)	*d*N ^5^ (s.e)	*d*S^6^ (s.e)	*d*N/*d*S	Tajima's *D*
*pkdhfr*	1–649	113	188	0.983 (0.002)	0.006 (0.001)	0.003 (0.001)	0.018 (0.006)	0.167	-2.24 (*P*<0.001)
*pfdhfr*	1–613	2	3	0.244 (0.081)	0	0.001 (0)	0	-	-0.82 (*P*>0.05)

^1^ Number of polymorphic sites.

^2^ Number of haplotypes (standard deviation).

^3^ Haplotype diversity (standard error).

^4^ Nucleotide diversity across synonymous and non-synonymous sites (standard error).

^5^ Nucleotide diversity at non-synonymous sites (standard error).

^6^ Nucleotide diversity at synonymous sites (standard error).

In marked contrast to *pkdhfr*, only 2 *pfdhfr* sites displayed evidence of polymorphism within Sabah (108N and 59R were fixed in the population), both representing non-synonymous variants (*d*N = 0.001 and *d*S = 0). A total of 3 haplotypes were observed, with accordingly low haplotype diversity (Hd = 0.244). Tajima’s *D* value was -0.820 but the result was not significant, likely reflecting the limited informative variation.

### *Pkdhfr* mutations, parasitemia and risk of severe malaria

There was no association between the presence of any *pkdhfr* mutation and higher parasitaemia or risk of severe disease, including specifically for the most common mutations R34L, T91P or V149A. This included no relationship between those with *pkdhfr* mutations and severe malaria at either QEH (5/16 [31%] vs. 30/111 [27%], *p* = 0.72), or the district sites (7.5% vs. 11.3%, *p* = 0.557).

### Changes in proportion of *pkdhfr* mutations over time

For patients admitted to QEH the study duration (18^th^ September 2010 – 20^th^ June 2014) was divided into 5 x 9-month time-periods, with mutations detected in 26/92 (28%), 19/68 (28%), 17/44 (39%), 12/44 (27%) and 10/28 (36%) patients, respectively, during these times (*p* = 0.49). There was also no increase over time in the proportion of patients with the 2 most common mutations, R34L and T91P.

### Geographic distribution of *pkdhfr* genotypes

**Fig 1:**
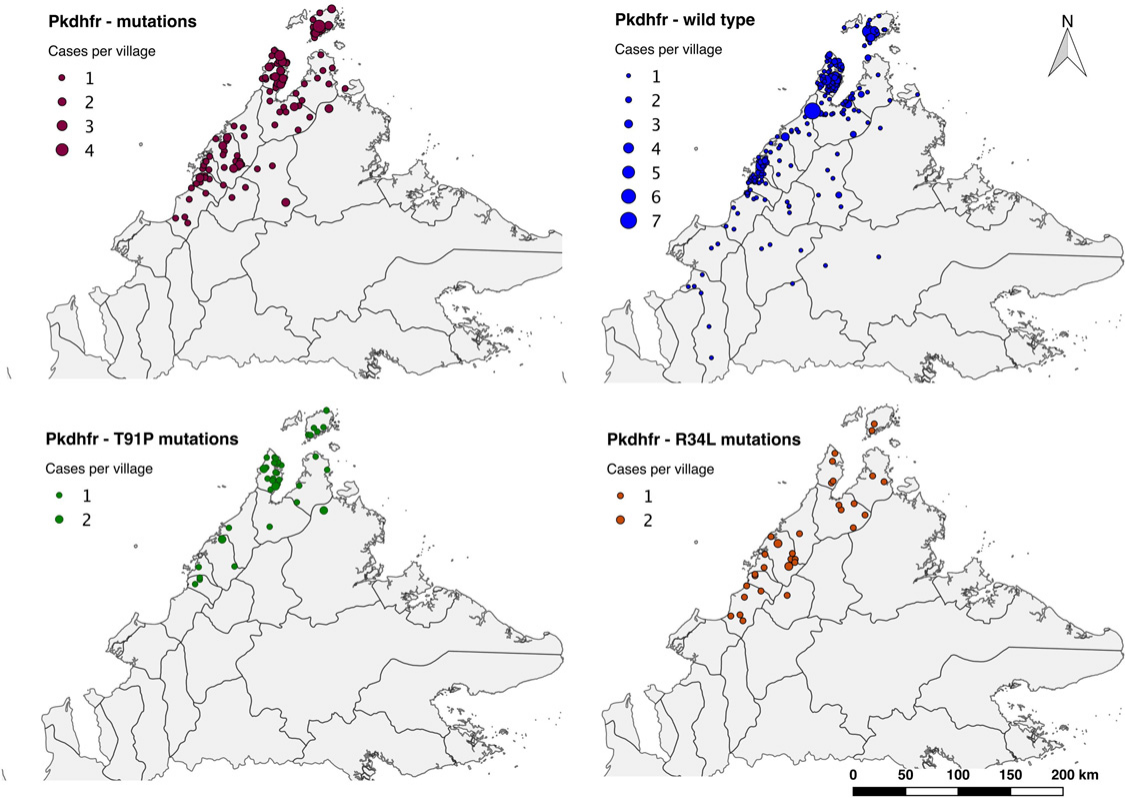
Relative distribution of *pkdhfr* genotypes from a tertiary and district referral hospitals in Sabah (with district administrative borders). *Image attached separately. a) All mutations. b) Wild-type. c) T91P mutations. d) R34L mutations.

Of all patients with *pkdhfr* sequencing (n = 449), 429 (96%) were successfully geo-located, including 138 (95%) patients with *pkdhfr* mutations and 291 (96%) with *pkdhfr* wild-type (**Fig 1**). The largest total number of patients mapped with both *pkdhfr* mutations (n = 64) and wild-type (n = 129) were from Kudat District. However, the highest proportion of *pkdhfr* mutations at a district level occurred in Pitas (10/13; 76%), followed by Ranau (6/8; 75%), and Penampang (11/15; 73%). Three districts did not record any *pkdhfr* mutations, however, these districts all had ≤7 patients with *pkdhfr* sequencing performed.

### Spatial and temporal clustering analysis

**Fig 2:**
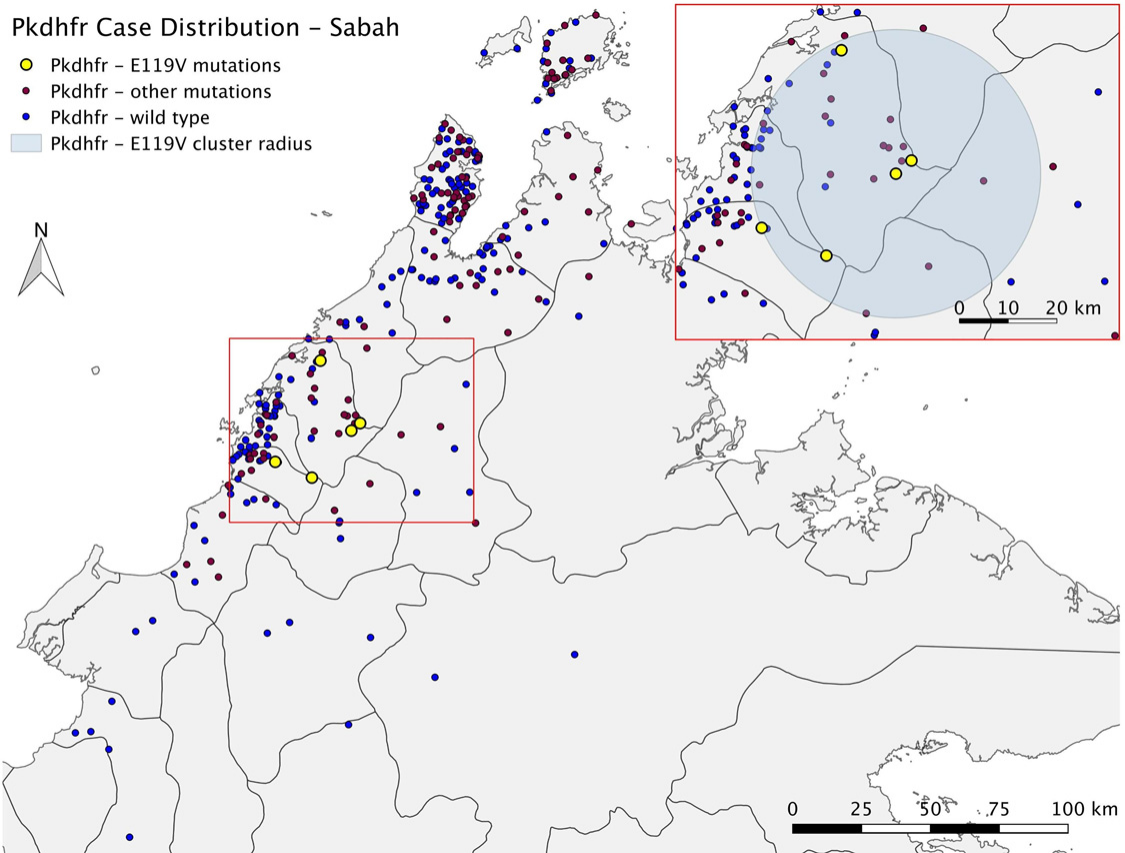
Baseline map containing inset with *pkdhfr* E119V mutation spatial cluster. *Image attached separately.

Of patients with *pkdhfr* mutations, a non-randomly distributed spatial cluster was detected among a subset of those with the R34L mutation (n = 9 [25%]; RR 8.94; *p* = 0.00051) (**Fig 1 and Table 5**). In addition, all patients mapped with an E119V mutation were found in a single cluster (n = 5 [100%]; RR>100; *p* = 0.0017) geographically overlapping with that of the R34L cluster **(Fig 2)**. None of the other *pkdhfr* mutations were detected in statistically significant spatial clusters, including the most common mutation, T91P (most likely cluster; RR 4.23; *p* = 0.296), or the *pkdhfr* wild-type as an independent group (most likely cluster; RR 1.51; *p* = 0.058).

**Table 5:** Spatial and temporal analysis–*pkdhfr* E119V and R34L mutation clusters.

Mutation	E119V			R34L		
Cluster type	Spatial	Space-time	Temporal	Spatial	Space-time	Temporal
Locations included	32	32	All	12	19	All
Radius	29.71 km	29.71 km		16.65km	19.97 km	
Time frame		7/2/2013–3/3/2014	3/1/2014–3/3/2014		20/3/2011–12/5/2012	27/4/2011–16/11/2012
Total number of cases	41	6	36	16	10	139
Cases with *pkdhfr* mutations	5	5	4	9	8	19
Expected number of mutations	0.48	0.07	0.42	1.31	1.08	13.28
Observed / expected	10.46	71.50	9.53	6.89	7.39	1.43
Relative risk	>100	>100	43.67	8.94	9.44	1.98
Log likelihood ratio	12.0283	24.5275	7.7004	13.2176	13.9163	2.1781
P-value	**0.0017**	**0.000007**	**0.009**	**0.00051**	**0.0075**	0.9800

Cases with a *pkdhfr* E119V mutation were the only group which demonstrated statistically significant clustering on temporal analysis (*p* = 0.009), with no significant temporal association seen for the two most common *pkdhfr* mutations T91P and R34L, or the wild-type. Clusters in both the *pkdhfr* R34L and E119V cases also appeared non-randomly distributed in the combined space-time analysis, with *p* values of 0.0075 and <0.0001, respectively.

### Epidemiology of *pkdhfr* E119V mutation cluster

Five patients with the E119V mutation were located within a 29.7 km radius, although all were from different villages. Median age was 49 years (IQR 25–29) and 3 were female (**Table 6**). All five patients presented within a 37-day period from January to February 2014, with two females presenting on the same day. Two patients had severe malaria, while the median parasite count for all patients was 5,034/μL (IQR 774–5384). All patients reported recent forest and/or plantation exposure.

**Table 6:** Patient details for *pkdhfr* E119V spatial cluster.

Case	Date enrolled	Fever duration	Address	Age	Sex	Occupation	Travel details	Lives within 20mins walk of forest and/or plantation	Recent overnight stay in forest and/or plantation
**1**	19/02/2013	7	Babagon	49	M	Farmer	Nil	Yes	Yes
**2**	22/01/2014	14	Pekan Nabalu	60	F	Fruit seller	Nil	Yes	No
**3**	22/01/2014	7	Nadau	25	F	Nil	Nil	Yes	No
**4**	14/02/2014	5	Togudon	22	M	Farmer	Nil	No	Yes
**5**	28/02/2014	3	Tinuhan	59	F	Housewife / rubber tapper	Nil	Yes	Yes

### Epidemiology of patients within the *pkdhfr* R34L mutation cluster

Nine patients with the R34L mutation were located within a 27.4 km radius. Median age was 55 years (IQR 51–68 years), five were male, and all were of either the closely related Dusun or Kadazan ethnicity. One patient had severe disease, with parasitaemia of 693,061/μL. Five reported a previous history of malaria. None were found to be family members, and it is not known if any of these patients had been exposed to one another. Two patients presented to the hospital 34 days apart between June and July 2011, while another three presented within a 44-day period between March and April 2012. All but one reported recent plantation and/or forest exposure.

### Effect of *pkdhfr* mutations on pyrimethamine binding

**Fig 3:**
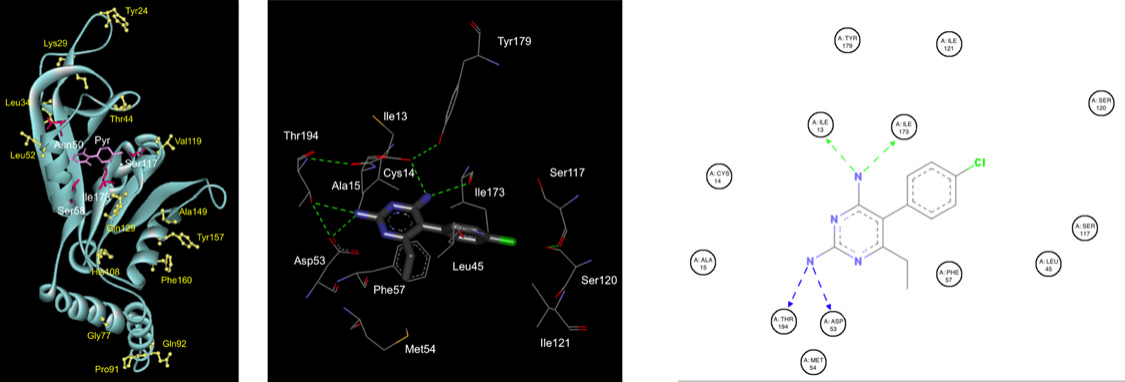
3D structural model of the *pkdhfr* 91P mutation and molecular docking of pyrimethamine in the active site. *Image panel attached separately. a) 3D structural model of *pkdhfr* with pyrimethamine bound at the active site. Mutations found in this study were shown as ball and stick coloured yellow. Equivalent residues of *pkdhfr* known to be related to pyrimethamine resistance in *pfdhfr* were presented as stick coloured dark pink and pyrimethamine shown as stick coloured magenta. b) Modelled *pkdhfr* binding site interaction with pyrimethamine. Pyrimethamine molecule was presented as stick and amino acid residues of *pkdhfr* were presented as line with carbon, nitrogen, oxygen and chloride colored as dark grey, blue, red and green, respectively. Hydrogen bonds are shown as green dashed line. c) 2D ligand interaction diagram of modelled pyrimethamine binding with all surrounding residues in active site of *pkdhfr* showing only contact with Ile13, Asp53, Ile 173 and Thr194. Hydrogen bonds are shown as dashed line. *Figure was generated by Discovery Studio Visualizer–Accelrys*.

The 3D structures of 19 *pkdhfr* amino acid sequences derived from patients with a *P*. *knowlesi* monoinfection were constructed using the SWISS MODEL server. Autodock vina was used to assess the effect of mutations on pyrimethamine binding. This encompassed both *pkdhfr* wild-type and all single monomorphic mutations (with the exception of D157Y), and also all genotypes with multiple mutations (with the exception of R34L-D157N). The results of molecular docking analysis indicated that five residues are in direct contact with the inhibitor pyrimethamine: I13, D53, S120 and I173. None of the mutations detected in this study were involved in inhibitor interactions and therefore, had no modelled effect on pyrimethamine binding **(Fig 3)**.

## Discussion

This study demonstrated a high rate of *pkdhfr* mutations in human knowlesi malaria, including a number of common mutations such as located at R34L, which along with E119V were associated with clusters of cases at adjacent villages. Clinical correlates hypothesized to be more likely with *P*. *knowlesi* H-H transmitted infections such as a higher likelihood of hyperparasitaemia and severe disease were not related to *pkdhfr* mutations[39,40]. The non-synonomous *pkdhfr* mutations described in this study do not provide evidence of H-H transmission, with homology modelling demonstrating that they are very unlikely to affect pyrimethamine binding and hence, are not likely to be a result of selective antifolate drug pressure.

Antifolate resistance can be selected readily in experimental malarias including simian *P*.*knowlesi* [67]. The utility of antifolate-resistance associated mutations as a marker of H-H transmission in this study requires both sufficient SP drug exposure in humans to select for resistant *P*. *knowlesi* parasite populations, and sustained transmission from humans to other human or monkey hosts. In the past *P*. *knowlesi* was often misdiagnosed as *P*. *malariae*[3,7], and therefore would have been nominally treated with chloroquine rather than SP according to longstanding Malaysian Ministry of Health guidelines. However, before the current pre-elimination malaria setting in Sabah where hospital admission is mandatory for all malaria cases, fever was frequently treated empirically with single dose SP by public health officials in the community for presumed non-severe malaria. The accumulation of *dhfr* mutations associated with SP-resistance in *P*. *falciparum* over 30 years of SP usage is well documented in Sabah[31,68,69]. With *P*. *knowlesi* endemic during the same period and commonly misdiagnosed as *P*. *falciparum*[3], the parasites infecting humans would have been exposed to some degree of within-host SP selection pressure [5,6]. For this to be selected at a population level would have required onward transmission.

One third of *P*. *knowlesi* isolates in this study had *pkdhfr* single point mutations, and 3% had double mutations. There were no triple mutants. The proportions of multiple mutants are less than predicted from a Poisson distribution arguing against stepwise selection. In contrast, in *P*.*falciparum* multiple *pfdhfr* mutations associated with antifolate resistance were found in this study, as previously described in Sabah, indicative of stepwise selection [31,68,69]. In *P*.*falciparum*, the primary *pfdhfr* mutation acquired in this progression is S108N, and in *P*. *vivax*, which is more closely related to *P*.*knowlesi* it is S117N. However, modelling showed residues involved in pyrimethamine binding in *pkdhfr* were located at I13, D53, S120, and I173, none of which were found in their mutant form in this study.

In this study, two *P*. *knowlesi* genotypes appeared to have non-random spatial distributions. This is consistent with a previous study demonstrating a higher level of genetic relatedness of *P*. *knowlesi* populations, as measured by the degree of multi-locus allelic frequencies (F_ST_ indices) from samples collected at different sites within Borneo, with decreasing geographical distance [44]. H-H transmission in these clusters appears unlikely but cannot be definitively excluded. Although the timing of hospital presentation for subsets of individuals in each cluster was consistent with potential exposure to a preceding human case with patent parasitaemia, the presence or duration of subpatent parasitaemia prior to this and hence the infectious period is not fully known. Finer scale clustering with H-H transmission would also be expected, including family or household members from the same village without significant travel history. Nearly all individuals in identified clusters also had forest and/or monkey exposure, and other potentially relevant geo-spatial variables were not controlled for in this analysis, while the relatively small number of samples in this study also limits their interpretation. The use of *P*. *knowlesi* spatio-temporal clusters alone to evaluate H-H transmission is constrained by the inability to discern whether a human was bitten by a human-fed mosquito, or if a cluster of humans were bitten by mosquitoes which had fed on the same monkey or monkey group. Assessing the possibility of individual level *P*. *knowlesi* H-H transmission using mutations associated with drug selection pressure, or other potentially better suited methods such as microsatellite markers or mitochondrial sequences, is also complicated by drug-exposed parasite populations in humans conceivably being transferred back to monkey hosts [17]. In this context comparative analysis of whether the same *P*. *knowlesi* dhfr mutations are present in nearby macaque hosts would also be useful but was not logistically feasible in this study.

The low level of nucleotide diversity and absence of putatively neutral synonymous variation in the *pfhdfr* region in Sabah is consistent with purifying selection acting upon a gene whose product has an important functional role, allowing little room for variation with the exception of the known pyrimethamine resistance conferring variants observed. In marked contrast, extensive haplotype and nucleotide diversity was observed in the *pkdhfr* region, with significantly higher diversity in synonymous versus non-synonymous sites. These patterns infer that the variation observed in *pkdhfr* might reflect neutral, stochastic processes, particularly within the monkey hosts where polyclonal infections are usual and transmission is intense[44]. Tajima’s *D* test also confirmed a significant excess of low frequency variants relative to expectation under neutral evolution consistent with other reports[44,70], potentially reflecting a recent population expansion, but not necessarily excluding purifying selection. A more comprehensive understanding of the genetic diversity across the *P*. *knowlesi* genome is needed for effective interpretation of the trends observed in *pkdhfr*. In addition, further analysis of the patterns of variation in *dhfr* in a broader range of human and simian *Plasmodium* species from settings with varying epidemiology and history of pyrimethamine use are required to elucidate the selective forces on *dhfr* in different *Plasmodium* spp.

## Conclusion

Despite an increasing incidence of *P*. *knowlesi* malaria in Sabah, Malaysia, the non-synonomous *pkdhr* mutations reported in this study do not appear to have been selected by repeated pyrimethamine exposure associated with competent onward transmission from humans, and hence cannot confirm H-H transmission. Despite these findings, low or unsustained H-H transmission cannot be excluded. In addition to establishing the transmission competency of naturally acquired *P*. *knowlesi* from human hosts, monitoring for spatio-temporal clusters of identical multi-locus genotypes and more detailed genome-wide studies may provide useful information on the likelihood of H-H transmission occurring.

## Supporting Information

S1 File *P*. *knowlesi* DHFR sequence data. (TXT)

## References

[1] Lee KS, Divis P. C., Zakaria SK, Matusop A, Julin RA, et al (2011) Plasmodium knowlesi: reservoir hosts and tracking the emergence in humans and macaques. PLoS Pathog 7: e1002015 10.1371/journal.ppat.1002015 21490952 PMC3072369

[2] Atkinson QD, Gray RD, Drummond AJ (2008) mtDNA variation predicts population size in humans and reveals a major Southern Asian chapter in human prehistory. Mol Biol Evol 25: 468–474. 10.1093/molbev/msm277 18093996

[3] Barber BE, William T, Grigg MJ, Yeo TW, Anstey NM (2013) Limitations of microscopy to differentiate Plasmodium species in a region co-endemic for Plasmodium falciparum, Plasmodium vivax and Plasmodium knowlesi. Malar J 12: 8 10.1186/1475-2875-12-8 23294844 PMC3544591

[4] Cox-Singh J, Davis TM, Lee KS, Shamsul SS, Matusop A, et al (2008) Plasmodium knowlesi malaria in humans is widely distributed and potentially life threatening. Clin Infect Dis 46: 165–171. 10.1086/524888 18171245 PMC2533694

[5] William T, Rahman HA, Jelip J, Ibrahim MY, Menon J, et al (2013) Increasing Incidence of Plasmodium knowlesi Malaria following Control of P. falciparum and P. vivax Malaria in Sabah, Malaysia. PLoS Negl Trop Dis 7: e2026 10.1371/journal.pntd.0002026.g006 23359830 PMC3554533

[6] William T, Jelip J, Menon J, Anderios F, Mohammad R, et al (2014) Changing epidemiology of malaria in Sabah, Malaysia: increasing incidence of Plasmodium knowlesi. Malar J 13: 390 10.1186/1475-2875-13-390 25272973 PMC4195888

[7] Singh B, Daneshvar C (2013) Human Infections and Detection of Plasmodium knowlesi. Clin Microbiol Rev 26: 165–184. 10.1128/CMR.00079-12 23554413 PMC3623376

[8] Marchand RP, Culleton R, Maeno Y, Quang NT, Nakazawa S (2011) Co-infections of Plasmodium knowlesi, P. falciparum, and P. vivax among Humans and Anopheles dirus Mosquitoes, Southern Vietnam. Emerg Infect Dis 17: 1232–1239. 10.3201/eid1707.101551 21762577 PMC3381379

[9] Putaporntip C, Hongsrimuang T, Seethamchai S, Kobasa T, Limkittikul K, et al (2009) Differential prevalence of Plasmodium infections and cryptic Plasmodium knowlesi malaria in humans in Thailand. J Infect Dis 199: 1143–1150. 10.1086/597414 19284284 PMC8817623

[10] Jongwutiwes S, Putaporntip C, Iwasaki T, Sata T, Kanbara H (2004) Naturally acquired Plasmodium knowlesi malaria in human, Thailand. Emerg Infect Dis 10: 2211–2213. 15663864 10.3201/eid1012.040293 PMC3323387

[11] Ng OT, Ooi EE, Lee CC, Lee PJ, Ng LC, et al (2008) Naturally acquired human Plasmodium knowlesi infection, Singapore. Emerg Infect Dis 14: 814–816. 10.3201/eid1405.070863 18439370 PMC2600232

[12] Luchavez J, Espino F, Curameng P, Espina R, Bell D, et al (2008) Human Infections with Plasmodium knowlesi, the Philippines. Emerg Infect Dis 14: 811–813. 10.3201/eid1405.071407 18439369 PMC2600254

[13] Jiang N, Chang Q, Sun X, Lu H, Yin J, et al (2010) Co-infections with Plasmodium knowlesi and other malaria parasites, Myanmar. Emerg Infect Dis 16: 1476–1478. 10.3201/eid1609.100339 20735938 PMC3294981

[14] Khim N, Siv S, Kim S, Mueller T, Fleischmann E, et al (2011) Plasmodium knowlesiInfection in Humans, Cambodia, 2007–2010. Emerg Infect Dis 17: 1900–1902. 10.3201/eid1710.110355 22000366 PMC3310675

[15] Moyes CL, Henry AJ, Golding N, Huang Z, Singh B, et al (2014) Defining the Geographical Range of the Plasmodium knowlesi Reservoir. PLoS Negl Trop Dis 8: e2780 10.1371/journal.pntd.0002780.s004 24676231 PMC3967999

[16] Vythilingam I (2010) Plasmodium knowlesi in humans: a review on the role of its vectors in Malaysia. Trop Biomed 27: 1–12. 20562807

[17] Chin W, Contacos PG, Collins WE, Jeter MH, Alpert E (1968) Experimental mosquito-transmission of Plasmodium knowlesi to man and monkey. Am J Trop Med Hyg 17: 355–358. 4385130 10.4269/ajtmh.1968.17.355

[18] Vythilingam I, Noorazian YM, Huat TC, Jiram AI, Yusri YM, et al (2008) Plasmodium knowlesi in humans, macaques and mosquitoes in peninsular Malaysia. Parasit Vectors 1: 26 10.1186/1756-3305-1-26 18710577 PMC2531168

[19] Jeslyn WPS, Huat TC, Vernon L, Irene LMZ, Sung LK, et al (2011) Molecular epidemiological investigation of Plasmodium knowlesi in humans and macaques in Singapore. Vector Borne Zoonotic Dis 11: 131–135. 10.1089/vbz.2010.0024 20586605 PMC3033207

[20] Eyles DE (1963) The species of simian malaria: taxonomy, morphology, life cycle, and geographical distribution of monkey species. J Parasitol 49: 866–887. 14084190

[21] Imai N, White MT, Ghani AC, Drakeley CJ (2014) Transmission and Control of Plasmodium knowlesi: A Mathematical Modelling Study. PLoS Negl Trop Dis 8: e2978 10.1371/journal.pntd.0002978.s005 25058400 PMC4109903

[22] Barber BE, William T, Dhararaj P, Anderios F, Grigg MJ, et al (2012) Epidemiology of Plasmodium knowlesi malaria in north-east Sabah, Malaysia: family clusters and wide age distribution. Malar J 11: 401 10.1186/1475-2875-11-401 23216947 PMC3528466

[23] Naidoo I, Roper C (2013) Mapping ‘partially resistant’, ‘fully resistant’, and “super resistant” malaria. Trends Parasitol 29: 505–515. 10.1016/j.pt.2013.08.002 24028889

[24] Flegg JA, Patil AP, Venkatesan M, Roper C, Naidoo I, et al (2013) Spatio-temporal mathematical modelling of mutations of the dhps gene in African Plasmodium falciparum. Malar J 12: 1–1. 10.1186/1475-2875-12-249 23866695 PMC3728261

[25] Peterson DS, Walliker D, Wellems TE (1988) Evidence that a point mutation in dihydrofolate reductase-thymidylate synthase confers resistance to pyrimethamine in falciparum malaria. Proc Natl Acad Sci U S A 85: 9114–9118. 2904149 10.1073/pnas.85.23.9114 PMC282674

[26] Plowe CV, Cortese JF, Djimde A, Nwanyanwu OC, Watkins WM, et al (1997) Mutations in Plasmodium falciparum dihydrofolate reductase and dihydropteroate synthase and epidemiologic patterns of pyrimethamine-sulfadoxine use and resistance. J Infect Dis 176: 1590–1596. 9395372 10.1086/514159

[27] Imwong M, Pukrittakayamee S, Looareesuwan S, Pasvol G, Poirreiz J, et al (2001) Association of Genetic Mutations in Plasmodium vivax dhfr with Resistance to Sulfadoxine-Pyrimethamine: Geographical and Clinical Correlates. Antimicrob Agents Chemother 45: 3122–3127. 10.1128/AAC.45.11.3122-3127.2001 11600366 PMC90792

[28] Imwong M, Pukrittayakamee S, Renia L, Letourneur F, Charlieu JP, et al (2003) Novel Point Mutations in the Dihydrofolate Reductase Gene of Plasmodium vivax: Evidence for Sequential Selection by Drug Pressure. Antimicrob Agents Chemother 47: 1514–1521. 10.1128/AAC.47.5.1514-1521.2003 12709316 PMC153323

[29] Tjitra E, Baker J, Suprianto S, Cheng Q, Anstey NM (2002) Therapeutic efficacies of artesunate-sulfadoxine-pyrimethamine and chloroquine-sulfadoxine-pyrimethamine in vivax malaria pilot studies: relationship to Plasmodium vivax dhfr mutations. Antimicrob Agents Chemother 46: 3947–3953. 12435700 10.1128/AAC.46.12.3947-3953.2002 PMC132782

[30] Hakim SL, Roohi SS, Zurkurnai Y, Rain AN, Mansor SM, et al (1996) Plasmodium falciparum: increased proportion of severe resistance (RII and RIII) to chloroquine and high rate of resistance to sulfadoxine-pyrimethamine in Peninsular Malaysia after two decades. Trans R Soc Trop Med Hyg 90: 294–297. 8758083 10.1016/s0035-9203(96)90258-8

[31] Cox-Singh J, Zakaria R, Abdullah MS, Rahman HA, Nagappan S, et al (2001) Short report: differences in dihydrofolate reductase but not dihydropteroate synthase alleles in Plasmodium falciparum isolates from geographically distinct areas in Malaysia. Am J Trop Med Hyg 64: 28–31. 10.4269/ajtmh.2001.64.1.11425158 11425158

[32] Dondero TJJ, Parsons RE, Ponnampalam JT (1976) Studies on the resistance of malaria to chloroquine and to a combination of chloroquine and pyrimethamine in Peninsular Malaysia. Trans R Soc Trop Med Hyg 70: 145–148. 785725 10.1016/0035-9203(76)90178-4

[33] Barber BE, William T, Grigg MJ (2013) A prospective comparative study of knowlesi, falciparum, and vivax malaria in Sabah, Malaysia: high proportion with severe disease from Plasmodium knowlesi and Plasmodium vivax but no mortality with early referral and artesunate therapy. Clinical Infectious Diseases. 10.1093/cid/cis902 23087389

[34] Iyer JK, Milhous WK, Cortese JF, Kublin JG, Plowe CV (2001) Plasmodium falciparum cross-resistance between trimethoprim and pyrimethamine. Lancet 358: 1066–1067. 10.1016/S0140-6736(01)06201-8 11589941

[35] Gregson A, Plowe CV (2005) Mechanisms of resistance of malaria parasites to antifolates. Pharmacological Reviews 57: 117–145. 10.1124/pr.57.1.4 15734729

[36] Carlton JM, Adams JH, Silva JC, Bidwell SL, Lorenzi H, et al (2008) Comparative genomics of the neglected human malaria parasite Plasmodium vivax. Nature 455: 757–763. 10.1038/nature07327 18843361 PMC2651158

[37] Hawkins VN, Joshi H, Rungsihirunrat K, Na-Bangchang K, Sibley CH (2007) Antifolates can have a role in the treatment of Plasmodium vivax. Trends Parasitol 23: 213–222. 10.1016/j.pt.2007.03.002 17368986

[38] Tyagi RK, Das MK, Singh SS, Sharma YD (2013) Discordance in drug resistance-associated mutation patterns in marker genes of Plasmodium falciparum and Plasmodium knowlesi during coinfections. Journal of Antimicrobial Chemotherapy 68: 1081–1088. 10.1093/jac/dks508 23292346

[39] Ciuca M, Chelarescu M, Sofletea A, Constantinescu P, Teriteanu E (1955) Contribution expérimentale á l’étude de l’immunité dans le paludisme. Ed Acad Rep Pop Roum: 1–108.

[40] Milam DF, Kusch E (1938) Observations on Plasmodium knowlesi malaria in general paresis. Southern Medical Journal 31: 947–949.

[41] Lim C, Hansen E, DeSimone TM, Moreno Y, Junker K, et al (2013) Expansion of host cellular niche can drive adaptation of a zoonotic malaria parasite to humans. Nat Commun 4: 1638–1639. 10.1038/ncomms2612 23535659 PMC3762474

[42] Barber BE, William T, Grigg MJ, Menon J, Auburn S, et al (2012) A prospective comparative study of knowlesi, falciparum and vivax malaria in Sabah, Malaysia: high proportion with severe disease from Plasmodium knowlesi and P. vivax but no mortality with early referral and artesunate therapy. Clin Infect Dis. 10.1093/cid/cis902 23087389

[43] Ahmed AM, Pinheiro MM, Divis PC, Siner A, Zainudin R, et al (2014) Disease Progression in Plasmodium knowlesi Malaria Is Linked to Variation in Invasion Gene Family Members. PLoS Negl Trop Dis 8: e3086 10.1371/journal.pntd.0003086.s017 25121807 PMC4133233

[44] Divis PCS, Singh B, Anderios F, Hisam S, Matusop A, et al (2015) Admixture in Humans of Two Divergent Plasmodium knowlesi Populations Associated with Different Macaque Host Species. PLoS Pathog 11: e1004888 10.1371/journal.ppat.1004888.s014 26020959 PMC4447398

[45] Grigg MJ, William T, Dhanaraj P, Menon J, Barber BE, et al (2014) A study protocol for a randomised open-label clinical trial of artesunate-mefloquine versus chloroquine in patients with non-severe Plasmodium knowlesi malaria in Sabah, Malaysia (ACT KNOW trial). BMJ Open 4 10.1136/bmjopen-2014-006005 PMC4139630 25138814

[46] Barber BE, William T, Grigg MJ, Menon J, Auburn S, et al (2013) A prospective comparative study of knowlesi, falciparum, and vivax malaria in Sabah, Malaysia: high proportion with severe disease from Plasmodium knowlesi and Plasmodium vivax but no mortality with early referral and artesunate therapy. Clinical Infectious Diseases 56: 383–397. 10.1093/cid/cis902 23087389

[47] Grigg MJ, William T, Drakeley CJ, Jelip J, Seidlein von L, et al (2014) Factors that are associated with the risk of acquiring Plasmodium knowlesi malaria in Sabah, Malaysia: a case-control study protocol. BMJ Open 4: 1–10. 10.1136/bmjopen-2014-006004 PMC4156811 25149186

[48] WHO (2010) Basic Malaria Microscopy. whqlibdocwhoint: 1–88. Available: http://whqlibdoc.who.int/publications/2010/9789241547826_eng.pdf. Accessed 10 September 2013.

[49] Padley D, Moody AH, Chiodini PL, Saldanha J (2003) Use of a rapid, single-round, multiplex PCR to detect malarial parasites and identify the species present. Ann Trop Med Parasitol 97: 131–137. 10.1179/000349803125002977 12803868

[50] Imwong M, Tanomsing N, Pukrittayakamee S, Day NPJ, White NJ, et al (2009) Spurious Amplification of a Plasmodium vivax Small-Subunit RNA Gene by Use of Primers Currently Used To Detect P. knowlesi. J Clin Microbiol 47: 4173–4175. 10.1128/JCM.00811-09 19812279 PMC2786678

[51] Hall TA (1999) BioEdit: a user-friendly biological sequence alignment editor and analysis program for Windows 95/98/NT Vol. 41. pp. 95–98.

[52] Pain A, Bohme U, Berry AE, Mungall K, Finn RD, et al (2008) The genome of the simian and human malaria parasite Plasmodium knowlesi. Nature 455: 799–803. Available: http://www.nature.com/doifinder/10.1038/nature07306. 18843368 10.1038/nature07306 PMC2656934

[53] Tajima F (1989) Statistical Method for Testing the Neutral Mutation Hypothesis by DNA Polymorphism. Genetics 123: 585–595. 2513255 10.1093/genetics/123.3.585 PMC1203831

[54] Nei M, Gojobori T (1986) Simple methods for estimating the numbers of synonymous and nonsynonymous nucleotide substitutions. Mol Biol Evol 3: 418–426. 3444411 10.1093/oxfordjournals.molbev.a040410

[55] Jukes TH, Cantor RC (1969) Evolution of protein molecules In: Munro HN, editor. Mammalian protein metabolism. New York: Academic Press pp. 21–132.

[56] Altschul SF, Gish W, Miller W, Myers EW, Lipman DJ (1990) Basic local alignment search tool. J Mol Biol 215: 403–410. 2231712 10.1016/S0022-2836(05)80360-2

[57] Larkin MA, Blackshields G, Brown NP, Chenna R, McGettigan PA, et al (2007) Clustal W and Clustal X version 2.0. Bioinformatics 23: 2947–2948. 10.1093/bioinformatics/btm404 17846036

[58] Bordoli L, Kiefer F, Arnold K, Benkert P, Battey J, et al (2009) Protein structure homology modeling using SWISS-MODEL workspace. Nat Protoc 4: 1–13. 10.1038/nprot.2008.197 19131951

[59] Kiefer F, Arnold K, Kunzli M, Bordoli L, Schwede T (2009) The SWISS-MODEL Repository and associated resources. Nucleic Acids Res 37: D387–D392. 10.1093/nar/gkn750 18931379 PMC2686475

[60] Brooker S, Clarke S, Njagi JK, Polack S, Mugo B, et al (2004) Spatial clustering of malaria and associated risk factors during an epidemic in a highland area of western Kenya. Trop Med Int Health 9: 757–766. 10.1111/j.1365-3156.2004.01272.x 15228485

[61] Laskowski RA, MacArthur MW, Moss DS, Thornton JM (1993) PROCHECK: a program to check the stereochemical quality of protein structures. Journal of applied crystallography 26: 283–291.

[62] Trott O, Olson AJ (2010) AutoDock Vina: improving the speed and accuracy of docking with a new scoring function, efficient optimization, and multithreading. J Comput Chem 31: 455–461. 10.1002/jcc.21334 19499576 PMC3041641

[63] Coleman M, Coleman M, Mabuza AM, Kok G, Coetzee M, et al (2009) Using the SaTScan method to detect local malaria clusters for guiding malaria control programmes. Malar J 8: 68 10.1186/1475-2875-8-68 19374738 PMC2679049

[64] Kulldorff M, Nagarwalla N (1995) Spatial disease clusters: detection and inference. Stat Med 14: 799–810. 7644860 10.1002/sim.4780140809

[65] Kulldorff M, Athas WF, Feurer EJ, Miller BA, Key CR (1998) Evaluating cluster alarms: a space-time scan statistic and brain cancer in Los Alamos, New Mexico. American journal of public health 88: 1377–1380. 9736881 10.2105/ajph.88.9.1377 PMC1509064

[66] Coatney GR, Collins WE, M W, Contacos PG (1971) The Primate Malarias. US Governtment Print Off. 366 pp.

[67] Singh J, Nair CP, Ray AP (1954) Studies on Nuri strain of P. knowlesi. V. Acquired resistance to pyrimethamine. Indian J Malariol 8: 187–195. 14353521

[68] Lim PK, Tan SK, Khoo AS, Noor Rain A, Nagappan S, et al (1998) The occurrence of dihydrofolate reductase (DHFR) point mutation (SER-108—>ASN-108) in Malaysian isolates of Plasmodium falciparum. Southeast Asian J Trop Med Public Health 29: 27–30. 9740263

[69] Lau TY, Sylvi M, William T (2013) Mutational analysis of Plasmodium falciparum dihydrofolate reductase and dihydropteroate synthase genes in the interior division of Sabah, Malaysia. Malar J 12: 445 10.1186/1475-2875-12-445 24321120 PMC4029390

[70] Assefa S, Lim C, Preston MD, Duffy CW, Nair MB, et al (2015) Population genomic structure and adaptation in the zoonotic malaria parasite Plasmodium knowlesi. Proc Natl Acad Sci USA: 201509534 10.1073/pnas.1509534112 PMC4620865 26438871

